# Presaccadic attention improves or impairs performance by enhancing sensitivity to higher spatial frequencies

**DOI:** 10.1038/s41598-018-38262-3

**Published:** 2019-02-25

**Authors:** Hsin-Hung Li, Jasmine Pan, Marisa Carrasco

**Affiliations:** 1 Department of Psychology, New York University, New York, New York USA; 2 Center for Neural Science, New York University, New York, New York USA

## Abstract

Right before we move our eyes, visual performance and neural responses for the saccade target are enhanced. This effect, presaccadic attention, is considered to prioritize the saccade target and to enhance behavioral performance for the saccade target. Recent evidence has shown that presaccadic attention modulates the processing of feature information. Hitherto, it remains unknown whether presaccadic modulations on feature information are flexible, to improve performance for the task at hand, or automatic, so that they alter the featural representation similarly regardless of the task. Using a masking procedure, here we report that presaccadic attention can either improve or impair performance depending on the spatial frequency content of the visual input. These counterintuitive modulations were significant at a time window right before saccade onset. Furthermore, merely deploying covert attention within the same temporal interval without preparing a saccade did not affect performance. This study reveals that presaccadic attention not only prioritizes the saccade target, but also automatically modifies its featural representation.

## Introduction

The visual system is limited by spatial resolution—the ability to discriminate two nearby points in space—that is highest at the fovea, the central area of the retina, and declines with eccentricity^1,2^. For that reason, when exploring visual scenes, human and primates perform large rapid eye movements–saccades–to bring objects of interest into the fovea. A central question in visual neuroscience is how visual perception seems so continuous and stable despite the fact that eye movements drastically shift the image in the retina.

Behavioral performance^3–9^ and perceived contrast^5^ for the saccade target is enhanced about 75–100 ms before saccade onset. Neurons in frontoparietal^10,11^ and early visual areas^12–16^ exhibit enhanced responses prior to saccades directed toward the neurons’ receptive fields, which may underlie such performance enhancement. This effect—presaccadic attention—is considered to prioritize the saccade target, improve visual performance^10,15^ and facilitate transaccadic perceptual stability^6,17^.

Presaccadic attention not only improves performance but also concurrently modulates the processing of featural information by sharpening orientation tuning^6,18^ and shifting spatial frequency (SF) tuning toward higher SFs^6^. However, the nature of these modulations is barely understood. To prioritize the saccade target and improve performance, presaccadic attention may modulate the processing of featural information in a flexible manner depending on stimuli content and task demands. Alternatively, presaccadic attention may modulate featural information in an inflexible manner. An inflexible modulation would occur automatically even when it could sometimes impair performance in a given task; however, in everyday vision, it would serve as a heuristic and a useful function that could improve performance most of the time. Here we investigated whether presaccadic attention modulates featural information, specifically SF, in a flexible or in an inflexible, automatic manner.

**Figure 1:**
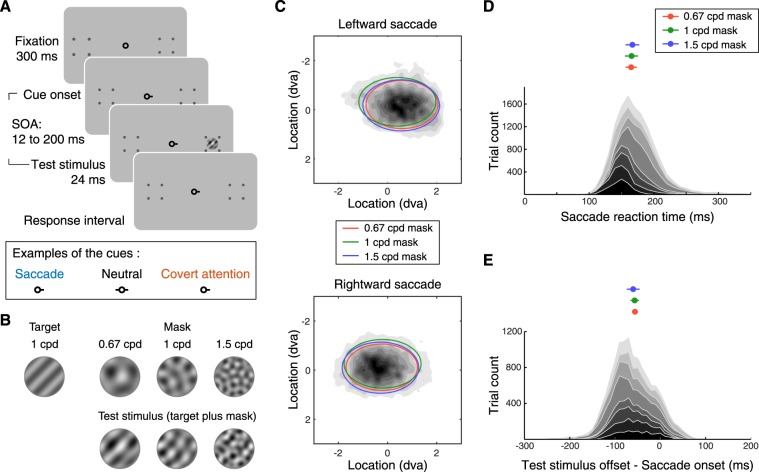
(**A**) Experimental procedure. Top row: Each trial started with a 300-ms fixation period, followed by the onset of the cue. The stimulus onset asynchrony (SOA) between the cue onset and stimulus onset was sampled uniformly between 12 to 200 ms. Bottom row: Examples of the cues used in each condition. In the saccade and covert-attention conditions, the cue pointed towards where the test stimulus would be presented. In the neutral condition, the cue pointed towards both locations. (**B**) Example of stimuli used in Experiment 1. Top row: The target was a 1 cpd grating tilted 45° clockwise or counterclockwise from vertical. The mask had three different SFs. Bottom row: The test stimulus was composed of the target superimposed on the mask. (**C**) Density of saccade landing position. The dark-gray shading represents group-averaged density of saccade landing position. The ellipses represent group-averaged data from different masking SFs. The ellipses are centered at the mean of saccade-landing positions. The width and height of the ellipses represent 1 standard deviation of (group-averaged) saccade-landing positions along the horizontal and vertical direction. (**D**) Bottom, distribution of trials sorted by saccade reaction time (delay between cue onset and saccade onset). Each shade of gray represents one individual. Top, group-averaged median saccade reaction time in each condition (masking SF). Error bars represent ± 1 s.e.m. (**E**) Bottom, distribution of trials sorted by the relative timing between saccade onset and the offset of the test stimulus. Each color represents one individual. Top, group-averaged median latency (relative timing between saccade onset and the test stimulus offset) in each condition (masking SF). Error bars represent ± 1 s.e.m.

In Experiment 1, we used a visual masking procedure and asked observers to discriminate the orientation (+45° vs. −45° off vertical) of a target (1 cycle per degree, cpd) presented 10° left or right of fixation in two conditions, presaccadic and neutral. The target signal was superimposed with masks that could suppress the responses for the target. Masks in three different SFs, lower than, the same as or higher than that of the target (Fig. 1), were used. We hypothesize that if presaccadic attention modulates SF processing flexibly, it would improve performance regardless of the masks’ SF. Alternatively, if presaccadic attention modulates SF processing by enhancing specific SF content automatically in an inflexible manner, it would either enhance or impair performance depending on the relation between the SF of the target and the SF of the mask. For example, if presaccadic attention enhances high-SF visual inputs inflexibly, presaccadic attention might result in a weaker enhancement or even an impairment on performance when the target was superimposed with a mask of higher SF content. This is because enhancing high-SF information would lead to a stronger suppression generated by the high-SF mask. To ensure that the results obtained in the presaccadic condition could not been merely explained by covert attention, we conducted a control experiment with the same timing as the presaccadic experiment, in which observers performed the same task while maintaining fixation. We note that this timing is too short for endogenous covert attention to be deployed^19–22^. However, the purpose of this experiment was not to investigate the effect of endogenous covert attention, but rather to rule out its possible contribution in the presaccadic condition.

To investigate whether the results we found in Experiment 1 can be generalized or are tied to specific target or masking stimuli, we conducted Experiment 2. In addition to investigating the effect of presaccadic attention by manipulating the SF of the mask while fixing the frequency of the target, in Experiment 2, we varied the SF of the target and fixed the frequency of the mask.

## Methods

### Participants

The experiment was conducted with the informed consent obtained from each observer. The University Committee on Activities involving Human Subjects at New York University approved the experimental protocols and all research was performed in accordance with relevant guidelines/regulations. Eight observers (19–31 years) participated in Experiment 1a. Seven of the eight observers from the Experiment 1a, along with one additional observer, participated in the control experiment (Experiment 1b) in which we tested the effect of covert voluntary attention. Nine observers (19–32 years) participated in Experiment 2.

### Setup

Observers sat in a dimly lit room with the chin rest positioned 57 cm from the monitor. The stimuli were presented on a gamma-correct monitor, with a resolution of 1280 × 960 pixels and a refresh rate of 85 Hz. MATLAB (Mathwork) with the Psychophysics Toolbox extensions^23^ was used to control the experiments. An EyeLink 1000 Desktop Mount (SR Research) monitored the gaze position of the right eye.

### Stimuli and procedure

#### Experiment 1a

Observers performed an orientation discrimination task, in which the test stimulus was composed of a target superimposed with a mask. The target was a 1-cpd sinusoidal grating. In each trial, the target was randomly oriented either +45° or −45° off vertical. The mask was generated by superimposing ten sinusoidal gratings (bases) of the same spatial frequency (SF). The orientations of the bases were set as ten even steps (starting from 0°) that uniformly tiled the orientation space. The phase of each basis was randomly chosen to take one of four values (0, *π*/2, π, 3*π*/2) in each trial. We tested masks of three different SFs: 0.67, 1 and 1.5 cpd. This type of mask has been used in previous psychophysical^24^ and neurophysiological^25^ studies. This design allowed the stimulus energy of the mask to be uniform in orientation space, but local and specific in SF space. The test stimulus, target plus mask, was presented in a circular aperture with a width of 3.8°. The edge of the circular aperture was smoothed by a raised-sinusoidal function. The two potential target locations, 10° left and right from the fixation (Fig. 1A), were marked by placeholders (each consisted of four dots forming a square) throughout the experiments.

Each trial started with a fixation period of 300 ms followed by the cue. In the saccade condition, the cue was a horizontal bar adjacent to the fixation point pointing to the location (left or right) where the test stimulus would be presented. Observers were instructed to saccade to the cued location as fast as possible. Shortly after the onset of the cue, the test stimulus (24 ms in duration) was presented at the cued location. The stimulus onset asynchrony (SOA) between the cue and the target was randomly sampled between 12 and 200 ms in each trial. This range of SOA was chosen because: 1. This interval allowed the test stimulus to be presented before saccade onset in most of the trials. 2. This interval was shorter than the time (about 300 ms) required for endogenous attention to be deployed (reviewed in^19^). In the neutral condition, the cue consisted of two bars pointing to both locations. Observers were required to maintain fixation at the central fixation point throughout each trial and eye position was monitored.

A response cue (an auditory tone) was played 700 ms after the offset of the test stimulus, which instructed observers to report the orientation of the target. Observers reported the target orientation by pressing one of two buttons on the left side of the keyboard (keys Z and X) when the test stimulus was presented at the left location, and one of two other buttons on the right right side (keys > and/) when the test stimulus was presented at the right location.

Observers first participated in a training session, which included a titration procedure and a set of training blocks, to familiarize them with the task. To determine the contrast of the mask and the contrast of the target for each observer, we conducted two sets of titrations: First, we titrated the contrast of the mask. In each trial, a patch of the 1-cpd mask (without the target) was presented at one of the two locations. We measured observers’ contrast detection threshold, defined as the contrast at which the observers could report the location of the mask with 75% accuracy. To ensure that the contrast of the mask was well above the threshold, we set the contrast of the mask to be 3.5 times the observers’ detection threshold for the rest of the experiment (the second titration and the main experiment). Second, we titrated the contrast of the target. We set the mask at the contrast level defined above, and measured observers’ contrast threshold of the target (set at 75% accuracy in the neutral condition) in discriminating the target orientation.

After the training sessions, observers completed the main experiment (>2000 trials in total) over 3–5 days. Each experimental session contained 6–8 blocks of 60 to 80 trials each. In each block, observers were tested with a fixed condition (saccade or neutral) and a fixed masking SF. The order of the conditions and masking SFs was randomized.

#### Experiment 1b

The control experiment included a covert-attention and a neutral condition. The covert-attention condition was very similar to the saccade condition in which a cue informed observers where the test stimulus will be presented; however, observers were required to maintain fixation at the central fixation throughout the trials.

#### Experiment 2

Here, for the main experiment, we fixed the SF of the mask at 1.5 cpd and tested targets in two different SFs (1 cpd and 2.25 cpd), one lower than the mask and one higher than the mask. Similar to Experiment 1a, two sets of titration procedures were run to determine the contrast of the mask and the contrast of the target. To titrate the contrast of the mask, we used a 1.5-cpd mask in a localization task. To titrate the contrast of the target, we used a 1.5-cpd target embedded in 1.5-cpd mask in an orientation discrimination task. All the other procedures of Experiment 2 were the same as those in Experiment 1a.

Note that throughout all the experiments, we blocked the stimuli (target SF and masking SF), and presented a demonstration of the test stimuli to be tested at the beginning of each block. This procedure reduced the chance that observers might perform poorly in the task due to the lack of prior knowledge about the test stimuli.

### Analysis

#### Performance

We computed observers’ performance in orientation discrimination by computing *d′*. We define a trial as a hit when the observer reported a right-tilted target to be right-tilted, and a trial as a false alarm when the observer reported a left-tilted target to be right-tilted. For the saccade condition, we binned the trials based on the time of stimulus offset relative to the time of saccade onset of each trial, and computed *d′* for the trials in a presaccadic time window (75 to 0 ms) before saccade onset.

We used a bootstrapping procedure to test whether performance between the saccade and neutral conditions differed significantly. For each combination of masking SF and time window, we used bootstrapping to generate a distribution of the difference score by the following procedure: For each observer, we resampled (with replacement) the trials from the saccade condition and the neutral condition separately, and computed *d′* from these resampled trials for each of the two conditions. We then computed the difference between these two *d′* as the individual difference score. The group-averaged difference score was computed by averaging the individual difference scores across all observers. The above procedures were repeated 2000 times (iterations) to generate the distribution of the group-averaged difference score, which was then used for the statistical test. We set the statistical criterion using a two-tailed test with Bonferroni correction for the number of SFs tested in each experiment. The p-value was computed as the proportion of the group-averaged difference scores that fell below or above zero.

We also used a boostrapping procedure to test the interactions between factors. For example, to test the interaction between SF and cue type, we employed the same procedure as above, but instead of computing the difference score, we computed the difference-difference score. Per iteration and per observer, we first computed the difference of *d′* between saccade and neutral condition for each masking SF as the difference scores, and then computed the difference between these difference scores across SFs. As we have three SFs in Experiment 1, we computed the difference-difference score for two pairs of SFs separately, one between 0.67-cpd mask and 1.5-cpd mask conditions, and one between 1-cpd mask and 1.5-cpd mask conditions. The group-averaged difference-difference score was computed by averaging the individual difference-difference scores across all observers. The above procedures were repeated 2000 times to generate the distribution of the group-averaged difference-difference score, which was then used for statistical testing.

#### Eye position

Eye position was monitored online. Trials with blinks or saccades (except for the response saccade required by the cue in the saccade condition) detected from the beginning of the trial to 200 ms after the offset of the test stimuli were aborted and repeated at the end of the block. We further analyzed eye position offline: raw eye position data were first smoothed with a Gaussian and we computed smoothed eye velocity using the eye positions of the five neighboring time points. Saccades were detected when the eye velocities exceeded the median velocity by 5 SDs for at least 8 ms^26^. Saccades separated by less than 10 ms were considered a single saccade. For the saccade condition, we analyzed the trials in which observers’ first response saccade occurred between 70–400 ms after the onset of the cue, and landed within the cued location with an error smaller than 2.5° from the center of the test stimulus.

After processing the data according to these criteria, we analyzed the data for the −75 to 0 ms presaccadic time window: about 720 total trials per masking SF (on average, 90 trials per observer and per masking SF) and about 855 total trials per target SF (on average, 95 trials per observer and per target SF).

## Results

In Experiment 1a, we tested whether the effect of presaccadic attention depends on the SF of the target and mask. We reported the statistical analysis using the boostrapping procedure (see Methods). We also performed an analysis of variance (ANOVA) and reported the results in Supplementary Information.

**Figure 2:**
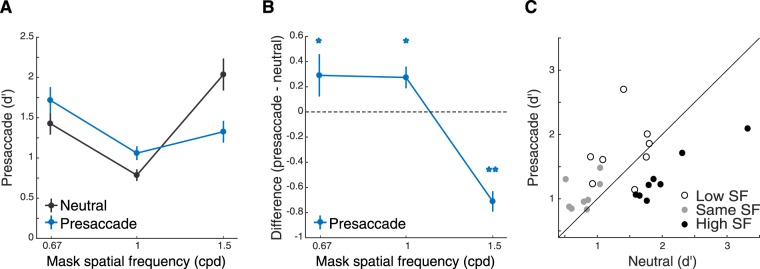
Experiment 1a. (**A**) Performance (*d′*) as a function of mask SFs. Error bars represent standard deviation of the bootstrapped distribution. (**B**) Difference of d’ between the saccade and neutral conditions. Saccade preparation improved performance for the low- and same-SF masks but decreased it for the high-SF mask. Error bars represent standard deviation of the bootstrapped distribution. *p < 0.05; **p < 0.005. (**C**) Scatter plot of performance of individual observers for each of the three masking SFs.

In the neutral condition, observers maintained fixation at the screen center throughout each trial. Performance (d′) exhibited a ‘V’ shape as a function of SF of the mask, being lower when the SF of the mask and target matched than when they mismatched (Fig. 2A, black lines). This pattern is consistent with masking functions that reflect the tuning property of the neural population underlying stimulus processing^27–30^.

In the saccade condition, a central pre-cue instructed observers to saccade to the upcoming target location (Fig. 1A; see Methods). Observers’ saccade reaction time (Fig. 1D) and saccade landing positions (Fig. 1C) were not affected by the SF of the stimuli. We first sorted the trials based on the relative timing between saccade onset and stimulus offset (Fig. 1E). To investigate the effect of presaccadic attention, we analyzed those trials in which the stimulus was presented right before saccade onset (−75 to 0 ms from saccade onset). This time window was chosen based on previous studies reporting significant effects of presaccadic attention on visual performance^5,6,31,32^. Compared to the neutral condition, performance improved in the saccade condition when the mask’s SF (0.67 cpd and 1 cpd) was lower than or the same as the target’s SF (1 cpd; Fig. 2) (bootstrapping test, *p* < 0.01, 95% CI [0.12 0.54] for 0.67-cpd mask, *p* < 0.05, 95% CI [0.09 0.47] for 1-cpd mask). In contrast, presaccadic attention impaired performance when the mask’s SF (1.5 cpd) was higher than the target’s SF (bootstrapping test, *p* < 0.005, 95% CI [−0.91–0.46] for 1.5-cpd mask). This frequency-dependent presaccadic attentional effect was confirmed by a significant interaction between masking SF and cue type (bootstrapping test on the interaction between SF and cue type; *p* < 0.005 for comparing the effect of presaccadic attention both between the 0.67-cpd and 1.5-cpd masks and between the 1-cpd and 1.5-cpd masks; ANOVA also revealed a significant two-way interaction between SF and cue type, see Table S1 in Supplementary Information).

Previous studies have shown that the effect of presaccadic attention builds up as a function of time before saccade onset^5,6,8,31,33^. We analyzed an earlier time window (-150 to -75 ms from saccade onset) and found that for the three masking SFs, performance (*d′*) followed this trend; it fell between that of the neutral condition and the late time window of the saccade condition. However, when comparing the performance in the earlier time window with those in the late time window and in the neutral condition, only the performance in the early time window was significantly worse than in the neutral condition for the 1.5-cpd mask (bootstrapping test, *p* < 0.005, 95% CI [−0.76–0.27]).

**Figure 3:**
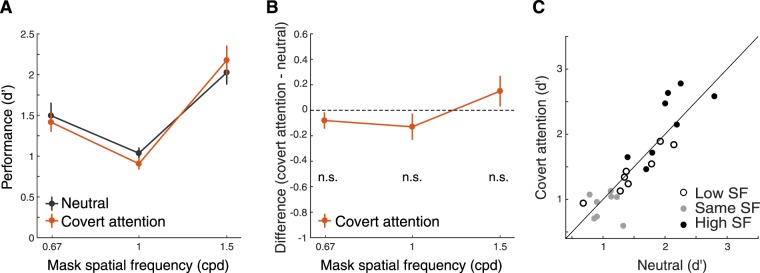
Experiment 1b. (**A**) Performance (*d′*) as a function of mask SFs in the Covert attention (control) experiment. Error bars represent standard deviation of the bootstrapped distribution. (**B**) Difference of *d′* between the covert attention and neutral conditions. Error bars represent standard deviation of the bootstrapped distribution. n.s. = no significant difference. (**C**) Scatter plot of performance of individual observers for each of the three masking SFs.

In the control experiment (Experiment 1b), we tested whether these effects could be due to the mere presence of the cue pointing to the target location or to endogenous covert spatial attention (the voluntary allocation of attention without concurrent eye movements^19^). In the covert-attention condition, we presented the same central pre-cue as in the saccade condition, but required observers to maintain fixation throughout each trial. We used the same time interval between the cue and the test stimuli as in Experiment 1a. The interaction between masking SF and cue type was not significant (bootstrapping test on the interaction between SF and cue type; *p* = 0.06 for comparing the effect of presaccadic attention between 0.67-cpd mask and 1.5-cpd mask, as well as for the comparison between the 1-cpd mask and 1.5-cpd mask; also see Table S2 in Supplementary Information for ANOVA). Performance did not differ between the covert-attention and neutral conditions for any of the mask SFs (two-tailed bootstrapping test; *p* = 0.72, 95% CI [−0.22 0.02] for 0.67-cpd mask, *p* = 0.14, 95% CI [−0.26 0.002] for 1-cpd mask, *p* = 0.16, 95% CI [−0.01–0.28] for 1.5-cpd mask; Fig. 3).

The results of this control experiment indicated that the SF-dependent modulations by presaccadic attention in Experiment 1a cannot be explained by covert endogenous attention. These results were confirmed by analyzing the data from the seven (out of eight) observers who participated both Experiments 1a and 1b. There was a significant three-way interaction for 3 masking SFs (0.67 cpd, 1 cpd and 1.5 cpd), 2 cue type (neutral and presaccade/endogenous attention), and 2 attention types (presaccadic attention and endogenous attention) (boostrapping test on the three-way interaction, *p* < 0.005 for 0.67-cpd and 1.5-cpd mask, *p* < 0.005 for the 1-cpd and 1.5-cpd mask; also see Table S3 in Supplementary Information for ANOVA).

In Experiment 1, we investigated the effect of presaccadic attention by manipulating the SF of the mask while fixing the frequency of the target. To test whether the effect we found depends on the SF of visual stimuli in general or whether it operates through target-specific (or mask-specific) filters, in Experiment 2 we varied the SF of the target (1 cpd and 2.25 cpd) and fixed the frequency of the mask (1.5 cpd).

**Figure 4:**
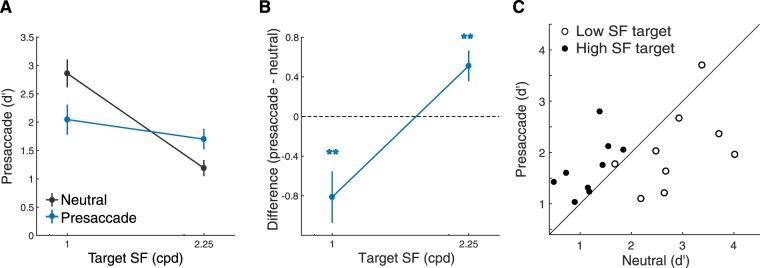
Experiment 2. (**A**) Performance (*d′*) as a function of target SFs. Error bars represent standard deviation of the bootstrapped distribution. (**B**) Difference of d’ between the saccade and neutral conditions. Error bars represent standard deviation of the bootstrapped distribution. *p < 0.05; **p < 0.005. (**C**) Scatter plot of performance of individual observers for each of the two target SFs.

We found a significant interaction between masking SF and cue type (Fig. 4; bootstrapping test on interaction between SF and cue type; *p* < 0.001; also see Table S4 in Supplementary Information for ANOVA). Compared to the neutral condition, performance improved in the saccade condition when the mask’s SF (1.5 cpd) was lower than the target’s SF (2.25 cpd) (bootstrapping test, *p* < 0.005, 95% CI [0.17 0.70]). In contrast, presaccadic attention impaired performance when the mask’s SF (1.5 cpd) was higher than the target’s SF (1 cpd) (bootstrapping test, *p* < 0.005, 95% CI [−1.17–0.59]). The results across the two experiments showed that whether presaccadic attention improved or impaired performance depends on the relation between the target’s SF and the mask’s SF, rather than a specific target (or masking) SF.

## Discussion

We recently showed that presaccadic attention concurrently improves performance and shifts the peak of SF tuning towards higher SFs^6^. In the saccade condition of that experiment, the peak of the tuning function shifted towards higher SFs, closer to the SF of the target. Such a shift could have emerged because observers employed a visual filter that better matched the target during the presaccadic interval or because SF tuning shifts inflexibly and automatically. The present results indicate that presaccadic attention enhances high-SFs inflexibly even when it is not beneficial for the task at hand.

Our previous study^6^ cannot dissociate these two ideas (flexible vs. automatic/inflexible) due to its experimental designs and results. In that study we used a reverse correlation approach by embedding the (1.5-cpd) target in a single type of mask. We observed that observers’ SF tuning functions peaked at a frequency lower than the target in the neutral condition. Presaccadic attention enhanced higher SFs and concurrently moved the peak of the SF tuning function to be more aligned with the target, thus improving performance in the task. Such results could have resulted from either a flexible enhancement of the SF of the target or an automatic enhancement of higher SFs. To dissociate these two possibilities, it is essential to investigate whether an enhancement of higher SFs would occur even when it impairs the performance.

The designs of the present experiments enable us to do so. The mask used in the previous study might have prevented any impairment to occur because its amplitude was flat in a SF range (1 to 2.25 cpd) that overlapped with the target (1.5 cpd). Here by using masks that were centered and concentrated at frequencies higher than the target, we created conditions in which automatically enhancing higher SFs would impair performance in orientation discrimination. In addition, by varying both masking SFs (Experiment 1) and target SFs (Experiment 2), we showed that presaccadic enhancement can be generalized, as it is not tied to a specific SF or image content.

The mask could suppress the neural responses to the target through divisive normalization^34^: the responses of the neurons selective for the target are divided by the responses of the population of neurons (the normalization pool), including those driven by the target and those driven by the mask. Based on divisive normalization, the neural response to the target can be conceptualized to be monotonically related to *C*_*t*_*/*(*C*_*t*_ + *C*_*m*_ + *σ*), in which the numerator is the target contrast, and the denominator is the sum of target contrast, mask contrast and a constant term^34^. The performance impairment, by the higher SF mask on a lower SF target, indicates that presaccadic attention preferentially enhances higher SFs. This enhancement may occur before divisive normalization and disproportionally inflate the *C*_*m*_ term (when the SF of the mask is higher than the SF of the target), resulting in stronger suppression on the target. This idea is consistent with the proposal that a preferential gain increment of visual neurons selective for higher SFs could be a common neural process underlying state-dependent modulations of SF processing^35,36^. Neurons selective for higher SFs also play a preferential role when exogenous attention^37–39^ and endogenous attention^40^ modulate visual perception.

We suggest that our results, enhancement or impairment of visual performance by presaccadic attention, can be generalized to visual inputs in general, and does not depend on the specific target (a grating) or mask (noise). In our experiments, we specifically defined a component of the test stimulus as the target and a component of the stimulus as the mask. Such design allowed us to create a condition in which enhancing higher frequencies would impair performance. Whether presaccadic attention improved or impaired performance depended on the relation between the SF of the target and the context (e.g. mask) in the task. For example, visual performance for a 1-cpd target was impaired by presaccadic attention in the presence of the 1.5-cpd mask in Experiment 1a, but was improved by presaccadic attention under the same 1.5-cpd mask in Experiment 2 when a 2.25-cpd target was used.

The results of the control experiment demonstrated that the mere presence of the cue, without observers executing a saccade to the target did not generate any SF specific effects compared to the baseline. The results also indicated that the perceptual modulations by presaccadic attention and endogenous covert attention can be dissociated. In line with previous studies^5,6^, our results demonstrated that presaccadic attention can take effect under a shorter time interval (12–200 ms SOA here) and its effects peak just before saccade onset. Endogenous covert attention requires longer time (~300 ms) to affect discrimination^19–22^. Note that we used a short timing here because the covert attention condition serves as a control experiment (Experiment 1b) for Experiment 1a. Here, we did not aim to investigate the effect of endogenous covert attention on SF processing under its optimal timing. With an optimal timing, endogenous covert attention modulates spatial resolution flexibly to improve performance; it does not enhance high-SFs when detrimental to the task^40,41^.

A recent study^42^ using an *averaging saccade* paradigm demonstrated another type of dissociation by showing that performance enhancement in the presaccadic interval occurred at the planned saccade location rather than at the actual saccade endpoint. Note that in our experiment, saccade endpoint coincided with the location of the test stimulus. Future studies could explore the spatial extent of the automatic high-SF enhancement by positioning the test stimulus at various locations relative to the saccade landing locations.

The effect of presaccadic attention on the processing of visual features like SF and orientation has only been studied recently. It has been shown that presaccadic attention reduces the orientation tuning width for the saccade target^6,18^, but not for the stimuli presented at the other locations^18^. The present study characterizes the effect of presaccadic attention on SF processing only at the saccade target. Future studies can employ a similar procedure and investigate the effect of presaccadic attention on SF processing across different locations.

Neurophysiological studies have reported neural correlates of presaccadic attention: Visual neurons selective for the saccade landing position increase their firing rate before a saccade^13–16^. However, very few studies have investigated the neural correlates of presaccadic attention on the processing of visual features^43^, which requires presenting stimuli varying along the featural dimension of interest. This approach has been used to study the relation between population neural responses and perceived stimulus positions during the peri-saccadic interval^44^. Future neurophysiological studies should explore other feature dimensions—like SF and orientation, which we have investigated psychophysically^6^—to further our understanding of the relation between eye movements and visual perception.

Saccades cause rapid and abrupt changes of retinal images. The brain maintains a clear percept of the visual world partly by suppressing visual inputs during saccades e.g.,^45,46^. Even so, humans are able to track task-relevant visual information around the time of the saccade^31,32,47^. Presaccadic attention may enable the visual system to hold on to the object of interest–the saccade target–by enhancing the responses^3–8^ for the saccade target right before the eye moves.

Going beyond prioritization and performance enhancement, our study highlights that automatic modulation of featural information is a critical function of presaccadic attention, as it occurred even at the expense of performance. What is the role of such an automatic inflexible modulation? Spatial resolution and high-SF sensitivity are highest at the fovea and decrease with eccentricity^48^. When executing a saccade, the image of the saccade target swiftly-and-drastically changes from a low-resolution peripheral representation to a high-resolution foveal image. The automatic enhancement of high-SF information revealed in the present study, as well as the orientation tuning^6,18^, at the saccade target may enable its presaccadic representation to be more similar to its post-saccadic (foveal) image, thus supporting visual stability by facilitating the integration between the pre- and post-saccadic visual inputs.

## Supplementary information

Supplementary Information
