# Supplementary material for: Presaccadic attention improves or impairs performance by enhancing sensitivity to higher spatial frequencies
Source: Sci Rep. 2019 Feb 25;9:2659. doi: 10.1038/s41598-018-38262-3 (PMC6389934; doi:10.1038/s41598-018-38262-3)
Supplement: Supplementary file 1 — Supplementary Information [file 41598_2018_38262_MOESM1_ESM.pdf]

Presaccadic attention improves or impairs performance by enhancing sensitivity to higher spatial frequencies

Hsin-Hung Li[1], Jasmine Pan[1] and Marisa Carrasco[1,2]

[1] Department of Psychology, New York University, New York, New York, USA
[2] Center for Neural Science, New York University, New York, New York, USA

Supplementary Information

Repeated Measures Two-Way Analysis of Variance Table

Source	*SS*	*df*	*MS*	*F*	*p*
Subjects	0.74	7			
Masking SF	5.57	2	2.79	10.29	0.002
Cue type	0.61	1	0.61	2.73	0.143
Masking SF x Cue type	1.18	2	0.59	6.53	0.009
Error	6.62	35	0.19		
Total	14.72	47			

Table S1. Experiment 1a. Presaccadic attention (constant SF target and different SF masks)

Repeated Measures Two-Way Analysis of Variance Table

Source	*SS*	*df*	*MS*	*F*	*p*
Subjects	2.79	7			
Masking SF	10.28	2	5.14	32.76	0.001
Cue type	0.01	1	0.01	0.12	0.742
Masking SF x Cue type	0.18	2	0.04	2.35	0.132
Error	3.02	35	0.09		
Total	16.27	47			

Table S2. Experiment 1b. Covert attention (constant SF target and different SF masks)

Repeated Measures Three-Way Analysis of Variance Table

Source	*SS*	*df*	*MS*	*F*	*p*
Subjects	2.50	6			
Masking SF	10.25	2	5.12	26.86	0.000
Cue type	0.00	1	0.00	0.01	0.935
Attention type	0.47	1	0.47	2.72	0.150
Masking SF x Cue type	0.45	2	0.23	9.53	0.003
Masking SF x Attention type	1.16	2	0.58	6.62	0.012
Cue type x Attention type	0.01	1	0.01	0.02	0.904
Masking SF x Cue type x Attention type	1.75	2	0.88	16.02	0.000
Error	6.10	66			
Total	22.70	83			

Table S3. Experiment 1. Presaccadic attention versus covert attention (constant SF target and different SF masks)

Repeated Measures Two-Way Analysis of Variance Table

Source	*SS*	*df*	*MS*	*F*	*p*
Subjects	9.28	8			
Masking SF	12.52	1	12.52	101.05	0.000
Cue type	0.35	1	0.35	1.32	0.285
Masking SF x Cue type	2.16	1	2.16	33.86	0.000
Error	3.62	24	0.15		
Total	27.93	35			

Table S4. Experiment 2. Presaccadic attention (different SF targets and constant SF mask).
